# Rapid Transcriptional Reprogramming Associated With Heat Stress-Induced Unfolded Protein Response in Developing *Brassica napus* Anthers

**DOI:** 10.3389/fpls.2022.905674

**Published:** 2022-06-09

**Authors:** Neeta Lohani, Mohan B. Singh, Prem L. Bhalla

**Affiliations:** Plant Molecular Biology and Biotechnology Laboratory, Faculty of Veterinary and Agricultural Sciences, The University of Melbourne, Parkville, VIC, Australia

**Keywords:** unfolded protein response (UPR), endoplasmic reticulum stress (ER stress), heat shock factors (HSF), heat shock proteins (HSP), plant reproduction, rapid response, anther, *Brassica*

## Abstract

Climate change associated increases in the frequency and intensity of extreme temperature events negatively impact agricultural productivity and global food security. During the reproductive phase of a plant’s life cycle, such high temperatures hinder pollen development, preventing fertilization, and seed formation. At the molecular level, heat stress-induced accumulation of misfolded proteins activates a signaling pathway called unfolded protein response (UPR) in the endoplasmic reticulum (ER) and the cytoplasm to enhance the protein folding capacity of the cell. Here, we report transcriptional responses of *Brassica napus* anthers exposed to high temperature for 5, 15, and 30 min to decipher the rapid transcriptional reprogramming associated with the unfolded protein response. Functional classification of the upregulated transcripts highlighted rapid activation of the ER-UPR signaling pathway mediated by ER membrane-anchored transcription factor within 5 min of heat stress exposure. KEGG pathway enrichment analysis also identified “Protein processing in ER” as the most significantly enriched pathway, indicating that the unfolded protein response (UPR) is an immediate heat stress-responsive pathway during *B. napus* anther development. Five minutes of heat stress also led to robust induction of the cytosolic HSF-HSP heat response network. Our results present a perspective of the rapid and massive transcriptional reprogramming during heat stress in pollen development and highlight the need for investigating the nature and function of very early stress-responsive networks in plant cells. Research focusing on very early molecular responses of plant cells to external stresses has the potential to reveal new stress-responsive gene networks that can be explored further for developing climate change resilient crops.

## Background

Extreme temperature events associated with climate change manifesting as heatwaves significantly threaten crop productivity and global food security (FAO, 2019). In particular, the excess heat events during the plant reproductive development can result in pollen sterility and loss of seed set (Rieu et al., 2017; Lohani et al., 2020; Lohani et al., 2021). Pollen development occurs inside the locules of anthers, starting with diploid microspore mother cells that undergo meiotic cell division resulting in four haploid microspores (Haerizadeh et al., 2006; Singh and Bhalla, 2007). Meiosis is tightly regulated genetically during pollen development (Golicz et al., 2021). The uninucleate microspores undergo two successive mitotic divisions to form mature trinucleate pollen grain. In most species, the onset of meiosis and pollen mitosis 1 seems most sensitive to environmental stresses (De Storme and Geelen, 2014).

Heat stress disturbs cellular protein-folding machinery causing an accumulation of misfolded/unfolded proteins, which can be toxic to cells. The stress-induced disturbance in protein homeostasis leads to the activation of protein quality control pathways, especially in the cytoplasm (cytosolic protein response, CPR) and endoplasmic reticulum (unfolded protein response, UPR) (Sugio et al., 2009; Buchberger et al., 2010; Liu and Howell, 2010; Lohani et al., 2022). A class of transcription factors (TFs) regulates CPR called heat shock transcription factors (HSFs) and involves the upregulation of heat shock proteins (HSPs) (Ohama et al., 2017). HSFs, under normal conditions, remain inactivated due to their interaction with HSPs (Hahn et al., 2011). However, this interaction is interrupted when unfolded/misfolded proteins recruit HSPs in the cytoplasm. The released HSFs translocate to the nucleus and bind to the heat shock elements (HSEs) present in the promoters of heat stress-responsive genes, thus driving their expression (Sakurai and Enoki, 2010; Guo et al., 2016).

In plants, two arms of the ER-UPR signaling pathway have been reported (Howell, 2013, 2021). One arm is mediated by INOSITOL REQUIRING 1-1 (IRE1) and bZIP60. Upon ER stress IRE1a and/or IRE1b dimerize, trans-auto-phosphorylate and catalyze the unconventional splicing of the transmembrane domain of *bZIP60* (Deng et al., 2011; Nagashima et al., 2011). The spliced *bZIP60* mRNA encodes a nucleus localized TF, which induces the transcription of ER-stress responsive genes (Iwata et al., 2008). Recently, in maize, bZIP60 was shown to activate an array of HSPs production, thereby revealing a key connection between the UPR in the ER and the CPR (Li et al., 2020). The other arm of the ER-UPR signaling pathway is mediated by bZIP28, a membrane-associated TF that is mobilized and transported to the Golgi in response to ER stress (Liu et al., 2007). In Golgi, two resident proteases (SITE-2 PROTEASE and an unknown protease) mediate proteolytic cleavage of bZIP28. The cleaved active form of bZIP28 translocates into the nucleus to activate the UPR gene expression (Iwata et al., 2017). Another dynamic transducer of ER-UPR is bZIP17 which is reported as functionally redundant with bZIP28 (Kim et al., 2018).

UPR is usually inactive in vegetative tissues in the absence of stress. There is evidence for its constitutive activity in the anther tissues (Deng et al., 2016). This UPR activation in anther tissues is comparable to activation of UPR during human B-cell differentiation, where UPR activation in plasma cells happens before the substantial synthesis and secretion of immunoglobulins (Shapiro et al., 2016). Even in the absence of external stress, an active UPR pathway is likely required to satisfy the high demands of secretory proteins throughout normal pollen development (Fragkostefanakis et al., 2016a; Singh et al., 2021). The trigger for the activation of UPR in anther tissues is not clear. Since hypoxia has been proposed as a potential trigger for acquiring meiotic cell fate by archesporial cells in the immature anthers (Kelliher and Walbot, 2012), it is likely, that hypoxic conditions are also responsible for activating UPR in anther tissues. Hypoxia-induced ER stress response has been reported as a pro-survival cellular adaptive mechanism in cancer and other pathologic conditions (Chipurupalli et al., 2019; Diaz-Bulnes et al., 2020).

While the transcriptional evidence exists for basal UPR activity in developing pollen for maintaining proteostasis for normal pollen development, less is known about the nature of reactive UPR activated in response to heat stress. Also, it is not known whether there are molecular differences in UPR responses in meiocytes and their post-meiotic progeny, the microspores. To address these questions, we exposed *Brassica napus* anthers containing meiocytes or microspores to heat stress for 5, 15, and 30 min. Here we give a brief perspective of the rapid transcriptional reprogramming of transcripts involved in unfolded protein response in ER and cytoplasm associated with the heat stress response of *Brassica napus* anthers at two stages of development.

## Experimental Results

### Response to Misfolded Proteins Is Rapidly Triggered in Developing Anthers Upon Heat Stress Exposure

**FIGURE 1:**
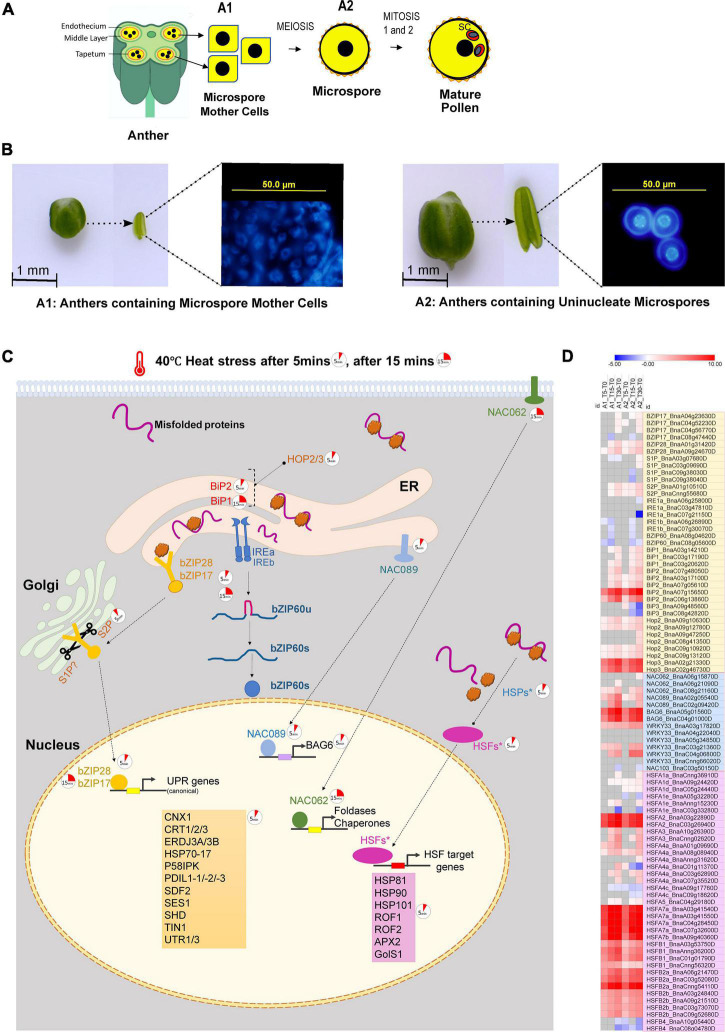
**(A)** A diagrammatic representation of sectioned anther showing microspore mother cells (A1 anther stage), microspore (A2 anther stage) and mature pollen. **(B)** *Brassica napus* anther samples collected for RNA-sequencing. Anthers containing microspore mother cells (A1) and anthers containing uninucleate microspores (A2) were collected immediately after 5, 15, and 30 min of high-temperature exposure at 40°C. **(C)** Rapid transcriptional reprogramming of genes associated with unfolded protein response in the ER and cytoplasm within 5–30 min of heat stress exposure of the anthers (A1 and A2); the 5 min clock icon indicates the upregulation of gene after 5 min of heat stress, and the 15 min clock icon indicates the upregulation of gene after 15 min of heat stress. **(D)** Heatmap showing the Log_2_fold change in expression of transcripts associated with regulation of unfolded protein response in the ER and cytoplasm in anthers containing microspore mother cells and anthers containing uninucleate microspores subjected to heat stress for 5, 15, and 30 min; the transcripts in the *light yellow box* highlight the transcripts regulating the two arms of the unfolded protein response in ER, i.e., the *bZIP28/17* dependent pathway and the *bZIP60-IREa/b* dependent pathway; the transcripts in the *light blue box* are ER-UPR associated *NAC* TFs and their selected targets; and the transcripts in the *light magenta box* are heat shock transcription factors (*HSFs*) which are associated with the regulation of unfolded protein response in the cytoplasm.

In the present study, *B. napus* plants bearing secondary inflorescences were exposed to a high temperature of 40°C. The anthers containing microspore mother cells (referred to as “A1” hereafter) and anthers containing uninucleate micropores (referred to as “A2” hereafter) (Figures 1A,B) were collected immediately after 5, 15, and 30 min of heat stress exposure. A1 and A2 were also collected from non-stressed *B. napus* plants. Three biological replicates were used for each treatment. Each replicate of two stages (A1 and A2) either consists of anthers containing microspore mother cells (A1) from 20 to 25 buds of size > 0.7 mm from ten plants or anthers containing uninucleate microspores (A2) from 10 to 15 buds of size 1–1.5 mm from ten plants. Differential expression analysis identified rapid, extensive transcriptional reprogramming within 5 min of heat stress with 5,901 and 10,692 genes identified as differentially expressed (fold change cutoff = ± 1.5, adjusted *p*-value < 0.01) in A1 and A2, respectively (Supplementary Figure 1). Functional classification of the DEGs identified the “response to protein folding” along with “response to heat” as the most significantly enriched upregulated GO biological process categories across all time points (Supplementary Figure 2A, Up-regulated GO Terms). KEGG pathway enrichment analysis identified “Protein processing in ER” as the most significantly enriched pathway (Supplementary Figure 2B), pointing toward the activation of unfolded protein response (UPR) as an immediate heat stress-responsive pathway during *B. napus* anther development.

### bZIP28 Arm as a Likely Rapid Responder in Unfolded Protein Response Signaling Pathway During Early Heat Stress Response in Anthers

*Brassica napus* (AACC, 2n = 4x = 38) is an allotetraploid species resulting from the hybridization of *B. rapa* (AA, 2n = 2x = 20) and *B. oleracea* (CC, 2n = 2x = 18) genomes approximately 7,500 years ago (Chalhoub et al., 2014). The *Brassica* genus diverged from their diploid progenitor *Arabidopsis thaliana* approximately 14–20 million years ago. Since then, the *Brassica* species underwent a whole-genome triplication event, followed by gene duplications, gene loss, and rearrangements. These genomic rearrangements resulted in differences in the gene copy numbers between *B. napus* and its diploid progenitors. To identify the *B. napus* genes involved in UPR signaling, we performed a genome-wide survey for selective identification of the *B. napus* genes homologous to the well-known ER stress-responsive *Arabidopsis* genes (Singh et al., 2021; Sze et al., 2021).

The homologs were further categorized based on their significant differential regulation (adjusted *p*-value < 0.01) across the contrasts (Supplementary Tables 1, 2). It is critical to highlight that these genes were identified as early heat stress-responsive genes only based on the differential expression of their respective transcripts. Additionally, the *B. napus* genes have been assigned putative gene names based on functional annotation analysis (BLASTp against *Arabidopsis* proteome, gene descriptors using PANNZER2 annotation tool and orthology analysis between *B. napus* and *Arabidopsis*). Further experimental validation is required to establish their functional roles in *B. napus*.

Upregulation of *bZIP28* (*BnaA09g24670D* in A1 and A2,*BnaA01g31420D* only in A2) transcripts indicates that the *bZIP28* arm of the UPR signaling was activated within 5 min in response to heat stress exposure. Compared to *bZIP28*, *bZIP17* homologs showed slight upregulation at 30 min in A1 and A2 (Figures 1C,D and Supplementary Table 1). Similarly, in locally heat-stressed *Arabidopsis* leaves, among *bZIP28* and *-17*, only *bZIP28* is upregulated within 2–8 min of heat stress exposure (Zandalinas et al., 2020). In contrast, the *IRE1*-*bZIP60* arm of UPR signaling was not upregulated during early heat stress response in A1 and A2. The expression profiles of *IREa/b* and *bZIP60* homologs in *B. napus* showed downregulation after 5–15 min of heat stress and slight upregulation after 30 min of heat stress (Figure 1D and Supplementary Table 1). In *Arabidopsis* leaves, 2–8 min of local heat stress exposure did not affect the levels of IREa/b transcripts, but bZIP60 was detected as an immediate heat stress responder (Zandalinas et al., 2020). In another transcriptome study, 3 h heat stress at 37°C did not affect bZIP17, bZIP28, bZIP 60, and IREa/b transcripts in *Arabidopsis* rosette leaves; however, all three bZIP TFs along with IREa transcripts were upregulated in early and late flowers (stage 1–9 and stage 10–12, respectively). Thus, the heat stress responsiveness of UPR signaling genes is dependent on the duration and intensity of heat stress and varies across different plant cell types.

Under non-stressed conditions, bZIP28 interacts with IMMUNOGLOBULIN-BINDING PROTEIN (BiP), which retains it on the ER membrane (Srivastava et al., 2013). In response to ER stress triggered by the accumulation of misfolded proteins, BiPs (BiP1/2/3) dissociate from bZIP28, and the released bZIP28 translocates to the Golgi apparatus. In both A1 and A2 stage *B. napus* anthers, the two *BiP2* homologs (*BnaA07g15650D*, *BnaC06g13860D*) were upregulated gradually at all-time points. In contrast, *BiP1 homologs* responded after 15 min of heat stress exposure (*BnaA03g14210D*, *BnaC03g20620D* in both A1 and A2 after 15 min, *BnaC03g17190D* in A2 after 15 min and in A1 after 30 min). Interestingly, in microspore mother cells containing anthers A1, BiP3 homologs (*BnaA09g48560D*, *BnaC08g42820D*) were not expressed under control or heat-stressed conditions. BiP3 homologs were expressed in haploid microspore containing anthers A2. However, their expression was downregulated upon exposure to heat stress. This observation in *B. napus* heat-stressed anthers contradicts the data reported in heat-stressed *Arabidopsis* reproductive tissues. Heat stress treatment (37°C for 3 h) of *Arabidopsis* reproductive tissues (flowers from stage 1–9 and stage 10–12) resulted in the upregulation of *BiP3* (Zhang et al., 2017). *BiP3* also showed rapid upregulation (within 2–8 min) in locally heat-stressed *Arabidopsis* leaves (Zandalinas et al., 2020). On the other hand, more prolonged heat stress exposure did not affect BiP3 transcripts in *Arabidopsis* leaves (Deng et al., 2016). The difference in *BiP3* expression levels between heat-stressed *B. napus* anthers and reproductive tissues in *Arabidopsis* is potentially due to differences in tissues analyzed and different heat stress treatments employed.

Furthermore, the basal expression of all *BiPs* under non-stressed conditions was higher in anthers containing microspores. In *Arabidopsis, bip1bip2bip3* triple mutation results in pollen abortion due to defective mitosis1 during microsporogenesis (Maruyama et al., 2014). A high expression level of BiPs in A2 anthers (Figure 1C) points to the significant conserved role of *BiPs* for microspore development in *Brassica napus*.

Following their translocation to the Golgi, bZIP28/17 are activated by proteolytic cleavage of their transmembrane domains mediated by Golgi-resident site proteases, S1P and S2P (Liu et al., 2007; Gao et al., 2008). *SITE-1 PROTEASE* (*S1P*) transcripts showed variable differential expression in A1 and A2 heat-stressed *B. napus* anthers. In the meiotic stage anthers (A1), there was no significant change in the level of transcripts encoding the S1P homologs. In microspore stage anthers (A2), two S1P homologs (*BnaA03g07680D* and *BnaC03g09690D*) transcripts were up-regulated after 30 min of heat stress. The other two S1P homologs (*BnaC09g38030D*, *BnaC09g38040D*) were either non-significant or downregulated. Contrary to S1P, in meiosis stage anthers, A1, *S2P* (*BnaA01g10510D*, *BnaCnng55680D*) transcripts were upregulated after 15 min of heat stress. In the A2 stage, S2P proteases were upregulated even after 5 min of heat stress exposure. The upregulation of *bZIP28/17* and the Golgi resident protease S2P transcripts further supports the activation of this arm of UPR signaling as a rapid heat stress response of *B. napus*. The cytosolic-facing components of bZIP17/bZIP28 are released from the Golgi membrane by S2P, allowing them to be transported into the nucleus to upregulate the expression of stress-responsive genes (Liu and Howell, 2010).

**FIGURE 2:**
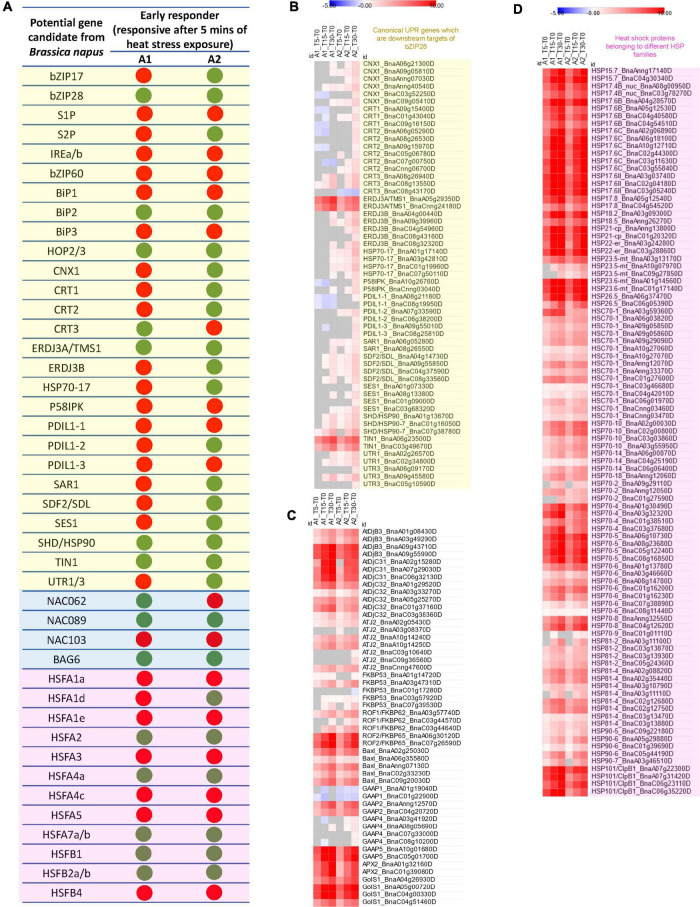
**(A)** Early heat stress-responsive genes involved in the regulation of UPR (transcripts regulating the two arms of the unfolded protein response in ER, i.e., the *bZIP28/17* mediated pathway and the *bZIP60-IREa/b* mediated pathway as well as canonical ER-UPR genes which are downstream targets of bZIP28*; light yellow box*) in ER, ER-UPR associated *NAC* TFs and their selected targets (*light blue box*) and cytoplasm (HSFs; *light magenta box*) in anthers containing microspore mother cells (A1) and anthers containing uninucleate microspores (A2) subjected to heat stress for 5 min. The green dot represents the 5 min of heat stress-induced change in expression of the gene, and the red dot represents no significant change in gene expression after 5 min of heat stress (Note: The potential gene candidate in *B. napus* was identified as an early heat stress-responsive gene if even one of the gene homologs displayed a significant change in expression, adjusted *p*-value < 0.01). Heatmaps showing the Log_2_fold changes in transcript expression of **(B)** Canonical UPR genes which are downstream targets of bZIP28 (*light yellow box*), **(C)** foldases, co-chaperones, *BAX Inhibitor-1* (*BAXI-1*), Golgi-apoptotic proteins (*GAAPs*), *Galactinol synthase1* (*GolS1*) and *Ascorbate peroxidase2* (*APX2*), and **(D)** heat shock proteins belonging to different *HSP* families (*light magenta box*).

Next, we identified the differentially regulated *B. napus* genes homologous to the canonical UPR genes well-curated in the literature associated with ER-stress response. Additionally, based on the available literature, we selected the genes reported as potential downstream targets of bZIP28 in *Arabidopsis*. Our data indicated that *B. napus* genes homologous to *BiP1*, *BiP2*, *BiP3*, *CALNEXIN 1 (CNX1)*, *CALRETICULIN (CRT) 1/2/3*, *THERMOSENSITIVE MALE STERILE 1 (TMS1/ERDJ3A)*, *ERDJ3B*, *HSP70-17*, *PROTEIN DISULFIDE ISOMERASE-LIKE (PDIL) 1-1/1-2/1-3*, *P58IPK*, *STROMAL CELL-DERIVED FACTOR 2-LIKE PROTEIN PRECURSOR (SDF2)*, *SENSITIVE TO SALT1 (SES1)*, *SHEPHARD (SHD/HSP90-7)*, *TUNICAMYCIN INDUCED 1 (TIN1)*, and *UTR1/3* (Figures 2A,B and Supplementary Table 2A) are potential gene targets of bZIP28.

*ERDJ3A/TMS* (*BnaA05g29350D*, *BnaCnng24180D*), an essential gene for male gametophytic thermotolerance in *Arabidopsis* (Yang et al., 2009), was the most significantly upregulated J domain co-chaperone (DnaJ) gene within 5 min of heat stress in both A1 and A2 *B. napus* anthers. Calnexin (CNX) and Calreticulin (CRT) present in the ER bind transiently to newly synthesized glycoproteins as they pass through the ER. PDILs catalyze disulfide bond formation during the folding process. Additionally, the UDP-glucose transporters, UTR1 and UTR3, transport nucleotide-sugars (UDP-Glu) into the ER lumen required for glycosylation (Liu and Howell, 2016). Interestingly, *CNXI*, *CRT1/2/3* and *PDIL1-1/1-2/1-2* homologous showed significant upregulation in heat-stressed *B. napus* anthers containing microspores (A2) (Figure 2B and Supplementary Table 2A). *CNXI*, *CRT1/2/3* and *PDIL1-1/1-2/1-2* are rapidly upregulated in *Arabidopsis* leaves within 2–8 min of heat stress exposure (Zandalinas et al., 2020). CNX and CRT have critical and overlapping roles during *Arabidopsis* vegetative growth and male gametophyte development. The *cnx1cnx2crt1crt2crt3* mutant is lethal in *Arabidopsis*, while the *crt1crt2crt3* triple mutation background has a detrimental effect on pollen viability and pollen tube growth (Vu et al., 2017). Additionally, *B. napus* transcripts of a plant-specific ER stress gene *TIN1* (*BnaA06g23500D*, *BnaC03g49670D*) were strongly upregulated within 5 min of heat stress in both A1 and A2 (Figures 2A,B and Supplementary Table 2A). In *Arabidopsis*, pollen grains of *TIN1*-overexpressing plants exhibited abnormal surface morphology suggesting a developmental role of *TIN1* in the secretion of proteins and/or lipids during pollen development (Iwata et al., 2012).

### Activation of NAC TFs: Unique Plant-Specific Transducers of Unfolded Protein Response as Part of the Rapid Heat Stress Response in Anthers

In addition to conserved transducers, the UPR in plants involves ER-stress upregulated plant-specific transcription factors, NAC062, NAC089, and NAC103. These TFs regulate different sub-sets of stress-induced genes (Sun et al., 2013; Yang et al., 2014a,b). *NAC062* and *NAC089* encode type-II membrane proteins localized in the plasma membrane and ER membrane, respectively, while *NAC103* encodes a soluble protein found in the nucleus. The expression of *NAC103* homologous gene *BnaC03g50150D* was downregulated in A1 and A2 *B. napus* anthers after 30 min of heat stress (Figure 1D and Supplementary Table 1). In *Arabidopsis*, a transcriptional regulatory cascade has been described in which NAC103 conveys ER stress signals from bZIP60 to downstream UPR genes *via* a newly discovered ER stress cis-element (UPRE-III) (Sun et al., 2013). The transcript levels in heat-stressed *B. napus* anthers suggest that the IRE1-bZip60 UPR arm is not activated following 30 min of heat stress exposure, which may explain the downregulation of NAC103 transcripts after 30 min of heat stress.

ER stress triggers the release of NAC062 (NAC062c) and NAC089 (NAC089c) cytoplasmic NAC domains from the membrane, followed by their translocation to the nucleus to induce the expression of genes involved in protein folding and programmed cell death (PCD), respectively. In heat-stressed A1 and A2 *B. napus* anthers, *NAC062* transcripts (*BnaA06g15870D*, *BnaA06g21090D*, *BnaC08g21160D*) upregulated following 15 min of heat stress (Figure 1D and Supplementary Table 1). The canonical UPR genes *SHD/HSP90, BiP2, BiP3, CNX1, CRT1, PDIL1-1*, and *PDIL1-2* are also identified as potential downstream targets of NAC062 in *Arabidopsis*. *NAC062* is also a rapid heat stress-responsive gene in *Arabidopsis* leaves (Zandalinas et al., 2020).

NAC089, the ER membrane-anchored TF, plays a key role in regulating PCD by inducing the expression of key PCD genes (Yang et al., 2014a). One copy of the *B. napus* gene homologous to *NAC089* (*BnaA02g05540D*) upregulated within 5 min of heat stress exposure was among the early responders of heat stress in both A1 A2 *B. napus* anthers (Figure 1D and Supplementary Table 1). *B. napus* genes homologous to NAC089 target genes such as *WRKY33* (*BnaA03g17820D*) and *BCL-2-ASSOCIATED ATHANOGENE 6* (*BAG6, BnaA05g01560D*, *BnaC04g01000D*) were also significantly upregulated within 5 min of heat stress. They showed further gradual upregulation with heat stress exposure in A1 and A2 *B. napus* anthers (Figure 1D and Supplementary Table 1).

### Heat Stress During Early Anther Development Leads to Robust Induction of Programmed Cell Death Inhibitor Genes

Likely Rapid ResponderABX Inhibitor-1 (BAXI-1), a conserved ER-localized suppressor of ER stress-induced PCD, modulates the threshold for cell death activation in response to diverse stressors, including excessive heat (Watanabe and Lam, 2008, 2009). In *Arabidopsis*, BAXI-1 expression is enhanced in response to various abiotic stimuli, including heat stress (Isbat et al., 2009; Watanabe and Lam, 2009; Zhang et al., 2017; Zandalinas et al., 2020). Overexpression of plant BI-1 has been shown to suppress abiotic stress-induced cell death in a variety of cells from yeast, plant (*Arabidopsis*, rice, rapeseed, and tobacco), and mammalian origins (Kawai-Yamada et al., 2001; Bolduc et al., 2003; Chae et al., 2003). Heat stress resulted in a rapid and robust increase in *B. napus BAXI-1* homologs (*BnaA02g25030D*, *BnaA06g35580D*, *BnaAnng07130D*, *BnaC02g33230D*, *BnaC09g20030D*) in A1 and A2 anthers. After 30 min of heat stress exposure, the expression levels were over 50 folds higher in heat-stressed vs. non-stressed anthers (Figure 2C and Supplementary Table 2B).

Golgi-apoptotic proteins (GAAPs) resident in membranes of Golgi complex confers resistance to a broad range of PCD inducers. Golgi localization of these proteins is unusual as apoptotic regulators are mainly localized in the cytosol, the ER and the mitochondria (Carrara et al., 2017). In our study, heat stress exposure for 5 min led to strong upregulation of *GAAP2* (*BnaAnng12570D*, *BnaC04g20720D*) and *GAAP5* (*BnaA10g01680D*, *BnaC05g01700D*) encoding genes in anthers at both anther developmental stages (Figure 2C and Supplementary Table 2B). *GAAP3* was not expressed in either non-stressed or heat-stressed *B. napus* anthers. In *Arabidopsis*, GAAP1, GAAP2, and GAAP3 all had redundant functions preventing cell death and delaying the ER stress-induced UPR activation (Wang et al., 2019). Our data thus highlights the potential role of *GAAP2* and *GAAP5* in regulating ER stress-induced cell death in *B. napus* heat-stressed anthers. Rapid upregulation of *GAAP2* and *GAAP5* reported in heat-stressed *Arabidopsis* leaves (Zandalinas et al., 2020) further suggested conserved rapid heat stress responsiveness of GAAP2 and GAAP5 Golgi-apoptotic proteins.

### Cytoplasmic Unfolded Protein Response Pathway Is Strongly Activated Within 5 Mins of Heat Stress in Anthers

The HSF-HSP complex mediates the unfolded protein response elicited due to the accumulation of misfolded proteins in the cytoplasm. The activation of HSPs by HSFs is identified as a classical heat shock response in plants. The HSF family in plants is much more complex and larger than any other eukaryote. *B. napus* has the largest reported HSF gene family (64 HSFs) (Zhu et al., 2017; Lohani et al., 2019). In A1 and A2 *B. napus* heat-stressed anthers, 19 out of 64 *HSFs* were strongly upregulated at all-time points (Figure 1D and Supplementary Table 1). Five minutes of heat stress upregulated *B. napus HSFA2*, *HSFA7a*, *HSFA7b* and *HSFB2a* homologs by > 100-fold (Figure 1D). *HSFA2*, *HSFA7a* and *HSFB2a* have been reported to be rapidly upregulated within 2–8 min of heat stress in *Arabidopsis* leaves (Oyoshi et al., 2020; Zandalinas et al., 2020). Furthermore, in *Arabidopsis* reproductive tissues, *HSFA2*, *HSFA7a*, *HSFA7b*, and *HSFB2a* were significantly upregulated after 3 h of heat stress (Zhang et al., 2017).

HSPs, the most prominent downstream target genes of HSFs, functioning as molecular chaperones, are responsible for protein folding, assembly, translocation, and degradation (Vierling, 1991). There are five primary families of HSPs in plants categorized based on their molecular weights- HSP100, HSP90, HSP70, HSP60, and small HSP (sHSP) (Wang et al., 2004). Heat stress-induced upregulation of all the HSP five major families in the A1 and A2 stages of *B. napus* anthers. Most *HSPs* are significantly upregulated within 5 min of heat stress (Figure 2D and Supplementary Table 1C). Their transcript levels gradually increased with exposure to heat stress in A1 and A2 anthers, making them rapid responders to heat stress during early male reproductive development in *B. napus*.

Furthermore, other than HSPs, HSFs regulate an array of genes involved in heat stress-responsive pathways. Among these genes is *ASCORBATE PEROXIDASE 2* (*APX2*), a critical reactive oxygen signaling pathway component. In heat-stressed *B. napus* anthers, A1 and A2, *APX2* (*BnaA01g32160D*, *BnaC01g39080D*) transcripts were significantly upregulated after 5 min of heat stress exposure (Figure 2C and Supplementary Table 2B). APX proteins are major antioxidant enzymes found in plants that detoxify H_2_O_2_ by reducing it with ascorbate. Heat stress triggers oxidative stress in plant cells, and upregulation of APX2 in response to heat stress may be required to scavenge reactive oxygen species (Panchuk Volkov and Schöffl, 2002; Miller et al., 2008; Zhang et al., 2017; Zandalinas et al., 2020). Another important downstream target of HSFs upregulated in heat-stressed anthers is the GALACTINOL SYNTHASE (GOLS1) enzyme. This enzyme is required to synthesize the raffinose family oligosaccharides (RFOs). RFOs act as osmoprotectants and antioxidants in plant cells (Panikulangara et al., 2004). *GolS1 homologous genes (BnaA04g26930D, BnaA05g00720D, BnaC04g00330D, BnaC04g51460D) in B. napus A1 and A2 anthers were also drastically upregulated after 5 min of heat stress. More than 100-fold upregulation was observed after 30 min of heat stress exposure* (Figure 2C and Supplementary Table 2B). *Similarly, GolS1 expression is significantly upregulated by heat stress in Arabidopsis vegetative and reproductive tissues* (Zhang et al., 2017; Zandalinas et al., 2020).

Co-chaperones and foldases are also critical elements of CPR (Sugio et al., 2009). HSP70 collaborates with co-chaperones, HSP40/DnaJ, to maintain protein homoeostasis. DnaJ proteins stimulate HSP70’s ATP hydrolysis, trapping the substrate (Kityk et al., 2018). The substrate protein then folds to its natural configuration upon release from HSP70. *B. napus* genes homologous to *DnaJ* genes such as *DjB3*, *DjC31/32* and *ATJ2* were significantly upregulated in both A1 and A2 within 5 min of heat stress exposure, with further enhancement of expression as the duration of heat stress increased (Figure 2C and Supplementary Table 2B). Heat stress also induced significant upregulation of *ROF1* (*FKBP62*) and *ROF2* (*FKBP65*) in A1 and A2 heat-stressed anthers (Figure 2C and Supplementary Table 2B). Compared to *ROF1 B. napus* homologs, the rapid upregulation of transcript levels was higher for *ROF2 B. napus* homologs. ROF1 regulates heat stress response by interacting with HSP90 and sustaining the expression levels of HSFA2 regulated sHSPs (Meiri and Breiman, 2009). In *Arabidopsis*, both *ROF1* and *ROF2* were upregulated by heat stress, with *ROF2* identified as a bonafide heat stress-responsive gene as it has almost undetectable expression under non-stressed conditions (Aviezer-Hagai et al., 2007). Similarly, under non-stressed conditions, *ROF2* transcripts (*BnaA06g30120D*, *BnaC07g26590D*) levels in A2 and A1 were very low (∼2 TPM) but increased drastically upon heat stress exposure (Supplementary Table 2B).

Another gene significantly upregulated in A1 and A2 anthers after 5 min of heat stress encode *HOP3* (*BnaA02g21330D*, *BnaC02g46730D*), an important cytosolic co-chaperone that mediates the HSP70-HSP90 interaction (Figure 1D and Supplementary Table 1). HOP function in plants is associated mainly with response to stress conditions (Torbio et al., 2020). In addition to its primary cytosolic localization, HOP3 also co-localizes with ER marker proteins (Torbio et al., 2020). *HOP3* gene is yet another rapid responder to heat stress in *Arabidopsis* vegetative tissue (Zandalinas et al., 2020). It is also reported to significantly upregulate in reproductive tissues in response to a longer heat stress treatment (Zhang et al., 2017). Notably, *Arabidopsis hop3* mutants exhibit decreased pollen germination and a hypersensitive phenotype in the presence of ER stress inducers (Fernández-Bautista et al., 2017), highlighting significant role of *HOP3* in pollen function.

## Summary and Future Directions

The knowledge of the nature and developmental stage and stress specificity of the rapid responses in plants is lacking due to the absence of studies on analyzing plant tissues at early time points following stress exposure (seconds-minutes scale) (reviewed in Zandalinas et al., 2020). There are no reports addressing the rapid heat stress response during reproductive development. Thus, here we present for the first time the early temporal transcriptome of heat-stressed anthers from two developmental stages to provide a perspective of the extensive transcriptional reprogramming and rapid high-temperature responsiveness of the genes associated with the unfolded protein response in ER and cytoplasm during early male reproductive development.

Our data reveal a rapid hyperactivation of hundreds of genes in the anther tissues in response to heat stress. Brief heat exposure of 5 min to anthers resulted in extensive transcriptional reprogramming with expression levels of some genes ramping up nearly fifty to a hundredfold compared to non-stressed controls. The first wave of rapid transcriptomic responses activated in 5 min involved genes associated with ER unfolded protein response and cytosolic unfolded protein response networks (Figures 1, 2). Such immediately activated genes have been classified as “primary response genes” that are “first responders” in the waves of transcription in response to a wide range of cell-extrinsic stimuli (Fowler et al., 2011). Further, it has been shown that such “first responder” genes do not require *de novo* protein synthesis as transcription factors necessary for their activation are already available in the cell (Fowler et al., 2011; Bahrami and Drablos, 2016; Guo et al., 2021). Such rapidly responding genes, particularly those involved in heat shock response, have been reported to be in the poised state with RNA polymerase II paused on the proximal promoter region (Vihervaara et al., 2018). Whether Pol II pausing occurs on transcriptional start sites of genes that show a drastic increase in the transcript levels with 5 min of heat shock treatment of *B. napus* anthers remains to be investigated. It is also significant that many of the genes showed rapid activation only in A2 anthers, making it likely that microspores are the expression site of some of these genes. Further, the robust transcriptional response of *GolS1*, a gene encoding *Galactinol Synthase1*, a key enzyme in the production of Raffinose and Stachyose sugars, points toward rapid metabolic reprogramming associated with changes in transcriptome during heat stress. Thus, multi-omics studies that complement transcriptional and metabolome profiling are required to unravel complex cellular responses to heatwave events.

## Data Availability Statement

The RNA-Seq datasets presented in this study are deposited at the NCBI Sequence Read Archive (BioProject ID: PRJNA841042, BioSample IDs: SAMN28573567 and SAMN28573568).

## Author Contributions

NL analyzed the sequencing data. All authors contributed to the writing of the manuscript and approved the submitted version.

## Conflict of Interest

The authors declare that the research was conducted in the absence of any commercial or financial relationships that could be construed as a potential conflict of interest.

## Publisher’s Note

All claims expressed in this article are solely those of the authors and do not necessarily represent those of their affiliated organizations, or those of the publisher, the editors and the reviewers. Any product that may be evaluated in this article, or claim that may be made by its manufacturer, is not guaranteed or endorsed by the publisher.
