# Supplementary material for: Rapid Transcriptional Reprogramming Associated With Heat Stress-Induced Unfolded Protein Response in Developing Brassica napus Anthers
Source: Front Plant Sci. 2022 Jun 9;13:905674. doi: 10.3389/fpls.2022.905674 (PMC9218420; doi:10.3389/fpls.2022.905674)
Supplement: Supplementary file 1 [file Data_Sheet_1.docx]

1. **Materials and methods**

**1.1 Plant material and heat stress treatment**

A medium/early maturing variety commercial cultivar of *Brassica napus* var. Garnet (AV Garnet) was selected for this research. *B. napus* var Garnet plants were grown in a Thermoline growth cabinet located within the Plant Growth Facility at the University of Melbourne, Australia. The plants were grown under controlled conditions at 23°C/18°C (day/night), 16 hours of light and 8 hours of darkness, light intensity at 200 μmolm^−2^s^−1^ and humidity at 60%. After 50-55 days after sowing (das) the plants that held secondary inflorescence were subjected to heat stress. Plants were stressed at 40℃ for 5, 15 and 30mins and immediately after heat stress exposure at a particular time point samples were collected. Three biological replicates were collected for each treatment. Anthers containing pollen/microspore mother cells (A1) were collected from 20-25 buds of size >0.7mm from ten plants for one biological replicate. Similarly, anthers containing uninucleate microspores (A2) from 10-15 buds of size 1-1.5mm from ten plants were collected per biological replicate and immediately stored in liquid nitrogen. This was followed by dissection of frozen buds without letting them thaw for more than 1 min. The dissection platform and forceps were sterilised and were kept submerged in liquid nitrogen. After dissection, the collected anthers were immediately stored in liquid nitrogen. DAPI staining (Otto, 1990) was performed to relate the size of the bud with pollen developmental stage. Three independent biological replicates were collected for each sample.

**1.2 RNA extraction, library preparation and RNA-Sequencing**

Total RNA isolation from both the non-stressed and heat stressed microspore samples was performed employing a mirVana™ microRNA isolation kit in accordance with the manufacturer instructions. These RNA samples were then stored in dry ice and shipped to BGI TECH SOLUTIONS (HONGKONG) CO., LIMITED where they were subjected to further testing in accordance with the company protocols for quality control for RNA sequencing. RNA sample integrity was tested employing an Agilent Bioanalyzer 2100™: this confirmed their suitability for being sequenced using the BGISEQ-500 platform for PE100 strand-specific mRNA sequencing; with a sequencing depth of 30 million raw reads for each sample. Following sequencing, data filtration was performed which included the removal of adaptor sequences, contamination, and poor-quality reads.

**1.3 Differential expression analysis**

FastQC v0.11.8 was employed to perform quality checks on the raw fastq files (Andrews, 2010). Reference transcriptome file for *Brassica napus* was downloaded from Genoscope (<http://www.genoscope.cns.fr/brassicanapus/data/>) (Chalhoub *et al.*, 2014). Quantification of transcript expression was performed using Kallisto v0.44.0 (Bray *et al.*, 2016). Conversion of transcript expression levels to gene expression levels was carried out using tximport v1.6.0 (countsFromAbundance="lengthScaledTPM") (Soneson *et al.*, 2015). Pre-filtering of low-count genes was undertaken by retaining solely those genes with >1 count in at least three samples. Differential expression analysis was performed using the voom method of the limma R package (Law *et al.*, 2014; Ritchie *et al.*, 2015). Principal component analysis (PCA) was undertaken for determining how related the biological replicates were. Batch effects were corrected for using the RUVSeq R package (Risso, 2015). The differential expression thresholds were established as log_2_ fold change of 0.585 and adjusted p-value cut off 0.01.

**1.4 Annotation and functional analysis**

Homologous *B. napus* genes, in comparison to the Arabidopsis proteome, were identified using the BlastP program with an E-value ≤ 1e-05. We further performed orthology analysis between *Brassica napus* and *Arabidopsis thaliana* genes using g:ortho a function of g:profiler (Raudvere *et al.*, 2019). We also transferred the gene descriptors as annotated by PANNZER2 tool to the differentially expressed genes (Törönen *et al.*, 2018).

The GO annotations for *B. napus* genes were downloaded from PlantRegMap (Tian *et al.*, 2020). The GO enrichment was performed using topGO (Rahnenfuhrer and Alexa, 2019). Subsequently, pathway annotation and enrichment analysis of DEGs was carried out using the KOBAS 3.0 database (Bu *et al.*, 2021). A GO term and a KEGG pathway were considered significantly enriched only when the corrected p-value for that pathway was <0.01 after applying Fisher's exact test and false discovery rate (FDR; BH method) correction, respectively.

**1.5 Data visualisation**

Visualization of significantly enriched functional pathways was performed by ggplot2 R package (Wickham *et al.*, 2016). R packed Complexheatmap package was used to generate heat maps of gene expression (Gu and Hübschmann, 2022). Transcript per kilobase million (TPM) was used for normalisation of gene-specific read counts and Z-score values i.e., scaled TPM values were used for representation of gene abundance in heatmaps. Morpheus software (https://software.broadinstitute.org/morpheus/) was used to generate heatmaps representing log_2_foldchange of differentially expressed genes.

**2. References**

**Andrews S**. 2010. FastQC: a quality control tool for high throughput sequence data. Babraham Bioinformatics, Babraham Institute, Cambridge, United Kingdom.

**Bray N, Pimentel H, Melsted P, Pachter L**. 2016. Near-optimal RNA-Seq quantification with kallisto. Nat Biotechnol **34**, 525-527.

**Bu D, Luo H, Huo P, Wang Z, Zhang S, He Z, Wu Y, Zhao L, Liu J, Guo J**. 2021. KOBAS-i: intelligent prioritization and exploratory visualization of biological functions for gene enrichment analysis. Nucleic acids research **49**, W317-W325.

**Chalhoub B, Denoeud F, Liu S, Parkin IA, Tang H, Wang X, Chiquet J, Belcram H, Tong C, Samans B**. 2014. Early allopolyploid evolution in the post-Neolithic Brassica napus oilseed genome. science **345**, 950-953.

**Gu Z, Hübschmann D**. 2022. Make Interactive Complex Heatmaps in R. Bioinformatics **38**, 1460-1462.

**Law CW, Chen Y, Shi W, Smyth GK**. 2014. voom: Precision weights unlock linear model analysis tools for RNA-seq read counts. Genome biology **15**, 1-17.

**Otto F**. 1990. DAPI staining of fixed cells for high-resolution flow cytometry of nuclear DNA. *Methods in cell biology*, Vol. 33: Elsevier, 105-110.

**Rahnenfuhrer A, Alexa A**. 2019. topGO: Enrichment analysis for gene ontology. R package version **2**.

**Raudvere U, Kolberg L, Kuzmin I, Arak T, Adler P, Peterson H, Vilo J**. 2019. g: Profiler: a web server for functional enrichment analysis and conversions of gene lists (2019 update). Nucleic acids research **47**, W191-W198.

**Risso D**. 2015. RUVSeq: remove unwanted variation from RNA-seq data. Bioconductor <https://bioconductor>. org/packages/release/bioc/html/RUVSeq. html.

**Ritchie ME, Phipson B, Wu D, Hu Y, Law CW, Shi W, Smyth GK**. 2015. limma powers differential expression analyses for RNA-sequencing and microarray studies. Nucleic acids research **43**, e47-e47.

**Soneson C, Love MI, Robinson MD**. 2015. Differential analyses for RNA-seq: transcript-level estimates improve gene-level inferences. F1000Research **4**.

**Tian F, Yang D-C, Meng Y-Q, Jin J, Gao G**. 2020. PlantRegMap: charting functional regulatory maps in plants. Nucleic acids research **48**, D1104-D1113.

**Törönen P, Medlar A, Holm L**. 2018. PANNZER2: a rapid functional annotation web server. Nucleic acids research **46**, W84-W88.

**Wickham H, Chang W, Wickham MH**. 2016. Package ‘ggplot2’. Create elegant data visualisations using the grammar of graphics. Version **2**, 1-189.
