# Supplementary material for: Rapid Transcriptional Reprogramming Associated With Heat Stress-Induced Unfolded Protein Response in Developing Brassica napus Anthers
Source: Front Plant Sci. 2022 Jun 9;13:905674. doi: 10.3389/fpls.2022.905674 (PMC9218420; doi:10.3389/fpls.2022.905674)

Figure S1. Number of differentially expressed genes in anthers containing pollen mother cells (A1) and anthers containing uninucleate microspores (A2) subjected to heat stress for 5, 15 and 30 mins

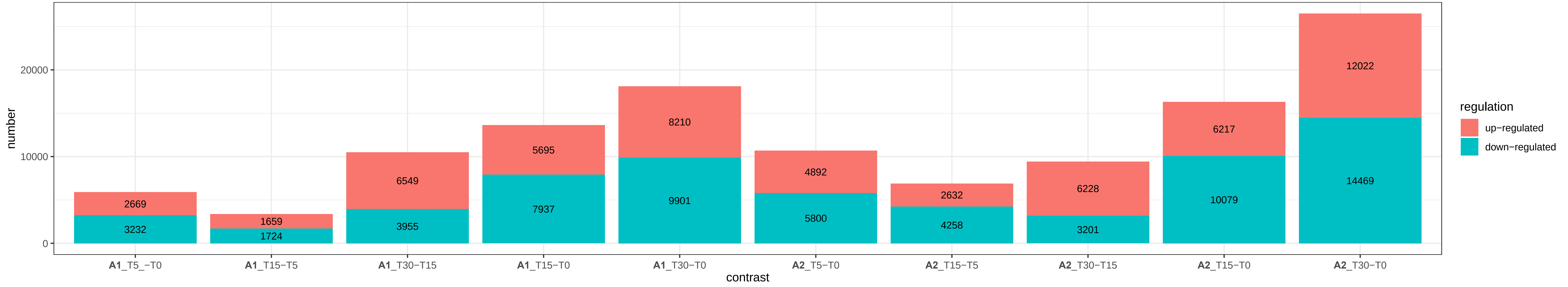

Supplement: Supplementary file 4 [file Image_1.PDF]
