# Supplementary material for: Rapid Transcriptional Reprogramming Associated With Heat Stress-Induced Unfolded Protein Response in Developing Brassica napus Anthers
Source: Front Plant Sci. 2022 Jun 9;13:905674. doi: 10.3389/fpls.2022.905674 (PMC9218420; doi:10.3389/fpls.2022.905674)

Figure S2. Functional annotation and enrichment analysis (A) Top significantly enriched GO terms associated with upregulated (left panel) and downregulated (right panel) differentially expressed genes in anthers containing pollen mother cells (A1) and anthers containing uninucleate microspores (A2) subjected to heat stress for 5, 15 and 30mins; (B) Top significantly enriched KEGG pathways associated with differentially expressed genes in anthers containing pollen mother cells (A1) and anthers containing uninucleate microspores (A2) subjected to heat stress for 5, 15 and 30mins.

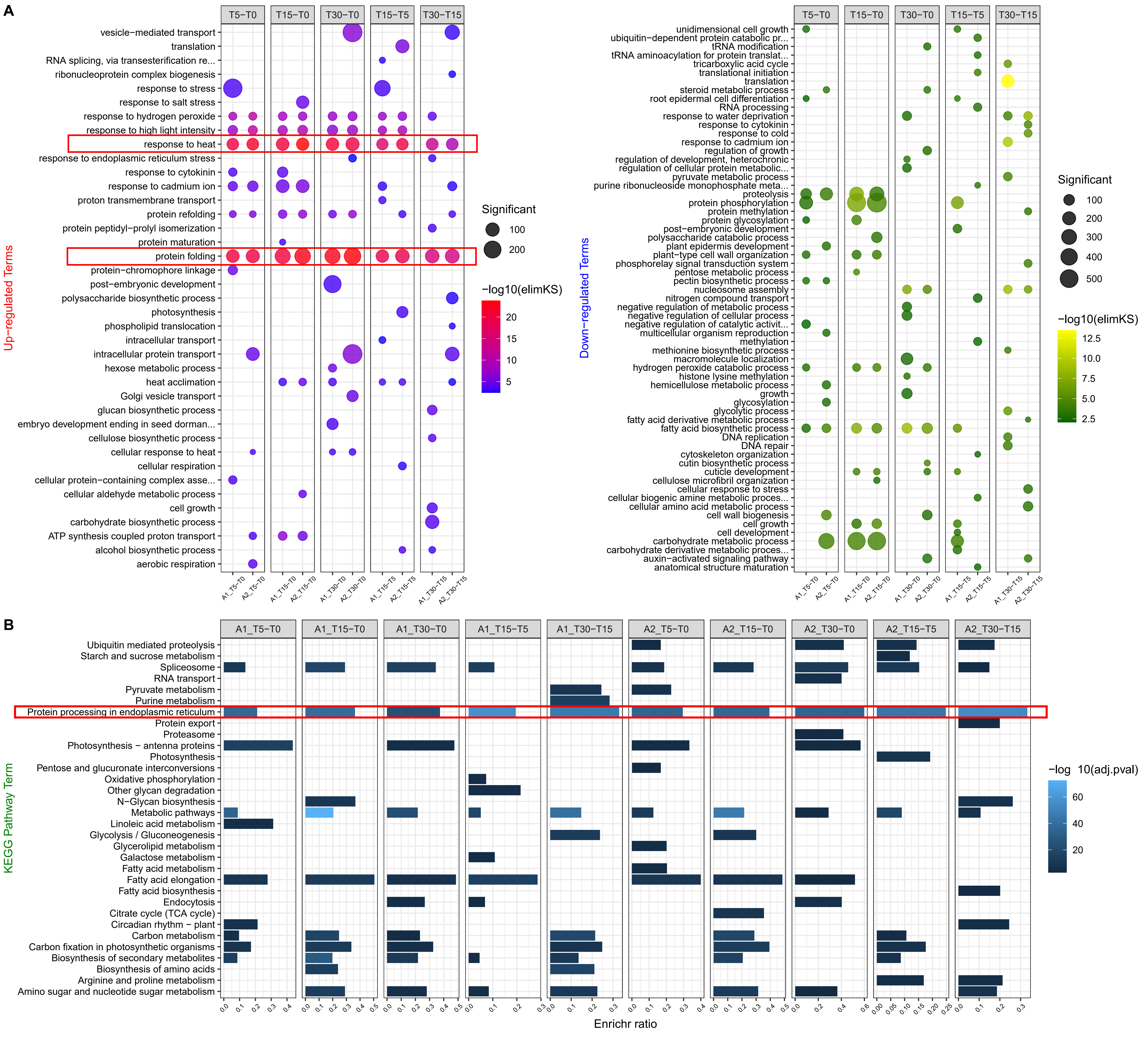

Supplement: Supplementary file 5 [file Image_2.PDF]
